# Exploring the mechanism of JiGuCao capsule formula on treating hepatitis B virus infection via network pharmacology analysis and *in vivo/vitro* experiment verification

**DOI:** 10.3389/fphar.2023.1159094

**Published:** 2023-06-08

**Authors:** Xu Cao, Ningyi Zhang, Hening Chen, Wei Wang, Yijun Liang, Jiaxin Zhang, Ruijia Liu, Shuo Li, Yuhao Yao, Qian Jin, Ziwei Guo, Yue Chen, Yuanyuan Gong, Xiaoke Li, Xiaobin Zao, Yong’an Ye

**Affiliations:** ^1^ Dongzhimen Hospital, Beijing University of Chinese Medicine, Beijing, China; ^2^ Liver Diseases Academy of Traditional Chinese Medicine, Beijing University of Chinese Medicine, Beijing, China; ^3^ Sun Simiao Hospital, Beijing University of Chinese Medicine, Tongchuan, China; ^4^ Key Laboratory of Chinese Internal Medicine of Ministry of Education and Beijing, Dongzhimen Hospital, Beijing University of Chinese Medicine, Beijing, China

**Keywords:** JiGuCao capsule formula, hepatitis B virus, network pharmacology, UHPLC-MS/MS, experimental verification

## Abstract

The JiGuCao capsule formula (JCF) has demonstrated promising curative effects in treating chronic hepatitis B (CHB) in clinical trials. Here, we aimed to investigate JCF’s function and mechanism in diseases related to the hepatitis B virus (HBV). We used mass spectrometry (MS) to identify the active metabolites of JCF and established the HBV replication mouse model by hydrodynamically injecting HBV replication plasmids into the mice’s tail vein. Liposomes were used to transfect the plasmids into the cells. The CCK-8 kit identified cell viability. We detected the levels of HBV s antigen (HBsAg) and HBV e antigen (HBeAg) by the quantitative determination kits. qRT-PCR and Western blot were used to detect the genes’ expression. The key pathways and key genes related to JCF on CHB treatment were obtained by network pharmacological analysis. Our results showed that JCF accelerated the elimination of HBsAg in mice. JCF and its medicated serum inhibited HBV replication and proliferation of HBV-replicating hepatoma cells *in vitro*. And the key targets of JCF in treating CHB were CASP3, CXCL8, EGFR, HSPA8, IL6, MDM2, MMP9, NR3C1, PTGS2, and VEGFA. Furthermore, these key targets were related to pathways in cancer, hepatitis B, microRNAs in cancer, PI3K-Akt signaling, and proteoglycans in cancer pathways. Finally, Cholic Acid, Deoxycholic Acid, and 3′, 4′, 7-Trihydroxyflavone were the main active metabolites of JCF that we obtained. JCF employed its active metabolites to perform an anti-HBV effect and prevent the development of HBV-related diseases.

## 1 Introduction

Hepatitis B virus (HBV) infection leads to chronic hepatitis B (CHB), which has become a serious global health concern. According to data from the World Health Organization (WHO), 30.4 million individuals have been diagnosed with hepatitis B infection, and 0.82 million people will pass away from diseases brought on by HBV infection in 2019 (WHO, 2021). Unfortunately, only 4.5 million of the estimated 27 million persons with positive HBV surface antigen (HBsAg) are suitable for antiviral therapy (Hutin et al., 2018). In China, about 5%–6% of the general population was detected HBsAg positive, suggesting there are 70 million chronic HBV-infected individuals as well as 20–30 million CHB patients (Liu et al., 2019). About 40% of CHB patients would progress to liver cirrhosis (Nayagam and Thursz, 2019). Hepatocellular carcinoma (HCC) tends to come on by persistent HBV infection in Asian and African nations (de Martel et al., 2015). According to the European Association for the Study of the Liver’s 2017 report, the average annual incidence of HCC in CHB patients accompanied by cirrhosis is 2%–5%, and the 5-year cumulative incidence of liver decompensation is 20% (European Association for the Study of the Liver, 2017). Overall, chronic HBV infection and its complications have led to a heavy burden of disease.

So far, there are mainly 2 types of medication used in CHB including nucleoside analogs (NAs) and interferons (IFNs) (Nguyen et al., 2020). NAs prevent HBV pregenomic RNA (pgRNA) from being reverse transcribed, which causes HBV DNA levels to drop quickly (Yuen et al., 2018). According to earlier research (Janssen et al., 2005; Lai and Yuen, 2008; Marcellin et al., 2013), NAs have a significant impact on lowering HBV DNA to a low level in short-term treatment and lowering the risk of progression to cirrhosis and HCC in long-term treatment. However, this antiviral therapy is still a long trip due to NAs’ unavoidable drawbacks, including covalently closed circular DNA (cccDNA) suppression challenges, lifelong treatment, drug resistance, and toxicity, which creates an urgent need for alternative treatments for CHB (Nguyen et al., 2020). IFNs provide several advantages, including a shorter course of treatment and a greater rate of HBV e antigen (HBeAg) and HBsAg clearance. However, due to their severe side effects and ineffective prevention of HBV DNA replication, IFNs are not the first-line medications that are advised in the clinic (Tan et al., 2018).

Traditional Chinese medicine (TCM) has been used to treat liver diseases as an alternative therapy for a long time, and it has demonstrated exceptional success in treating chronic HBV infection in recent years (Tsai et al., 2015). It was reported that the ethanol extract from polygonum cuspidatum (also known as Hu-Zhang in China) could inhibit HBV *in vitro* at a concentration of 10 μg/mL (Peng et al., 2013). Su-duxing, a medicine extracted from a Chinese botanical drug, demonstrated strong inhibitory effects on both entecavir (ETV)-resistant and wild-type HBV (Liu et al., 2018). Oxymatrine could suppress HBV replication and eliminate blood HBsAg and intrahepatic HBcAg more effectively than ETV at 20 mg/kg (Sang et al., 2017). Chinese patent medication Liuweiwuling Tablet demonstrated a strong inhibitory effect on both wild-type and ETV-resistant HBV, which may be related to improving IFN-β and IFN-γ production (Ge et al., 2021). Extract of TCM formula Le-Cao-Shi (LCS) could suppress the production of HBsAg, HBeAg, and HBV-DNA in a duck hepatitis B model, and it might also lessen the levels of the aspartate aminotransferase (AST) and alanine aminotransferase (ALT) and improve the histological abnormalities in duck liver (Zhao et al., 2019). Additionally, TCM has a significant benefit in regulating blood ALT levels without producing significant adverse effects, which makes it useful in the treatment of CHB (Zhang et al., 2010). Thus, TCM could exert the anti-HBV effect in many ways like reducing HBsAg and HBeAg levels, suppressing HBV replication, enhancing host immunity, and so on.

In TCM theory, CHB is a complex-syndrome disease with the core syndrome as stagnation of Gan and deficiency of Pi qi, and damp-heat in the Gan and Dan (Ye et al., 2018). By this, the JiGuCao capsule formula (JCF), a type of TCM approved by the China National Medical Products Administration (approval number: Z20025742) (Wei et al., 2022), is utilized and demonstrates a positive effect on protecting the liver through dropping enzymes and a potential anti-virus effect (Sui et al., 2002; Lei, 2018c; Li et al., 2021). JCF contains 8 botanical drugs such as Abrus melanospermus subsp. melanospermus, Artemisia capillaris Thunb., Gardenia jasminoides J. Ellis, Panax notoginseng (Burkill) F.H.Chen, Paeonia lactiflora Pall., Origanum vulgare L., Lycium chinense Mill., and Ziziphus jujuba Mill., and 2 animal materials such as Bovis calculus artifactus and Suis Fellis Pulvis. And the standard information and hyperlinks of the above drugs were shown in Supplementary Table S1. JCF could catharsis Gan and Dan, remove heat, and eliminate poison, which accords with the core syndrome of CHB (He et al., 2022; Li et al., 2022). However, the underlying mechanism of JCF on CHB remains unclear.

**FIGURE 1:**
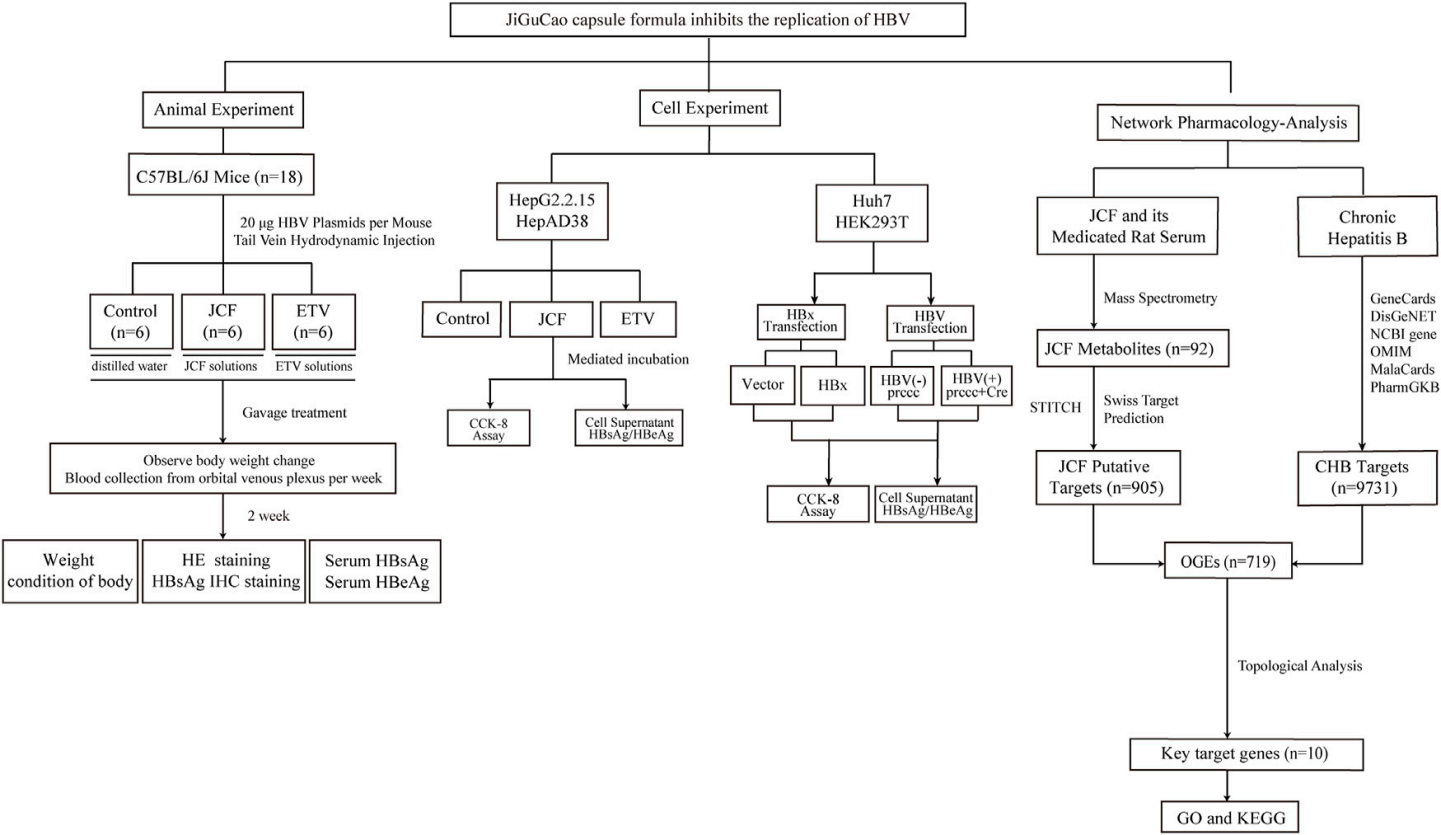
Flowchart of the study. We started by conducting *in vivo* experiments using the hepatitis B virus (HBV) replication mouse model. The mice’s weight, serum HBV s antigen (HBsAg), serum HBV e antigen (HBeAg), and pathological condition were detected. Next, cell experiments showed that the JiGuCao capsule formula (JCF) inhibited the expression of HBsAg, HBeAg, and HBV x protein (HBx) and the proliferation of HBV-expressing cell lines *in vitro*. On the right side of the network-pharmacology-analysis, we obtained the targets of JCF and chronic hepatitis B, and further constructed network analyses to get key target genes and key metabolites. Then we analyzed the enrichment of key target genes in databases.

Network pharmacology has recently been widely applied to explore the synergistic effect and mechanism of the TCM formula due to its research methodology, which helps to optimize the TCM formula (Ma et al., 2020; Cao t al., 2021). Additionally, the identification of TCM metabolites has made extensive use of ultra-high-performance liquid chromatography-tandem mass spectrometry (UHPLC-MS/MS) as an advanced detection method (Cao et al., 2021; Li et al., 2021). Our study aims to uncover the anti-HBV pharmacological mechanism of JCF through *in vivo* and *in vitro* experiments and investigate its metabolites by UHPLC-MS/MS. In Figure 1, the workflow for this study was shown.

## 2 Materials and methods

### 2.1 Design of experiment on HBV replication model in mice

20 specific pathogen-free (SPF) grade male C57BL/6J mice (weight 18 ± 2 g, 5-week-old) were purchased from Beijing Huafukang Bioscience Co., Ltd, and housed in the SPF animal laboratory of Dongzhimen Hospital under a standard 12/12 h light/dark cycle. The ambient temperature of the feeding environment is 23°C–25°C, and the relative humidity is 40%–60%. All animals received human care throughout the experiments, and the study protocols were approved by the Animal Research Ethics Board of Beijing University of Chinese Medicine Dongzhimen Hospital (Approval number: 21-46). We adopted an HBV replication mouse model via hydrodynamic injection (HDI) as previously described (Zao et al., 2021). 10 μg HBV-expressing plasmids were dissolved in 2 mL of phosphate buffer saline (PBS) (almost equivalent to 8% of the mouse body weight) and then injected into the tail vein of C57BL/6J mice within 5–8 s. And we next detected the serum HBsAg level of mice 2 days after injection to select HBV-positive mice. Then, we randomly divided the HBV-positive mice into three groups, one group was treated with sterile water by gavage (Control), one group was treated with JCF solution by gavage (JCF), and the last group was treated with ETV solution by gavage (ETV). According to clinical equivalent dosages in humans, we determined the doses for mice. JCF solution (780 mg/kg/d) was administered to the JCF group, ETV solution (0.065 mg/kg/d) was administered to the ETV group, and distilled water was administered to the Control group. JCF (Batch No.2102013) was provided by Guangzhou Kangchen Pharmaceutical Co., Ltd. ETV (E421565) was purchased from Shanghai Aladdin Biochemical Technology Co., Ltd. All drugs were given by gavage with a volume of 0.1 mL/10 g once a day for 2 weeks. During treatment, we recorded the level of HBsAg by collecting the serum of all mice and weighing them weekly. At the end of the experiment, mice’s livers were collected for the pathological section to carry out hematoxylin and eosin (H&E) staining and immunohistochemistry (IHC) detection of HBsAg.

### 2.2 Preparation of JCF medicated rat serum

20 male Wistar rats (weight 175 ± 20 g) were purchased from Beijing Vital River Laboratories Animal Technology Co., Ltd. The animal ethical approval number was 21–10, and the rats were housed under the same conditions as mice. The JCF-medicated serum group was administered intravenously with 1.08 g/kg/d of JCF solution, which was twice the clinically equivalent adult dose. The Control group was administered intravenously with distilled water. After 7 days, all rats were in abrosia for 6 h before the last administration. All animals were anesthetized with 3% pentobarbital sodium solution (0.2 mL/100 g) by intraperitoneal injection. After the abdominal aorta blood collection, the serum was separated by centrifuging at 3,000 r/min for 10 min. After inactivation at 56°C for 30 min, the serum was sterilized by filtration through a 0.22 μm microporous filter and stored in the −80°C refrigerator for later use.

### 2.3 UHPLC-MS/MS analysis of JCF and its medicated rat serum

The metabolites analysis of JCF and its medicated rat serum was conducted by Waters Synapt G2-Si Qtof high-resolution mass spectrometry and Unifi software at the Tsinghua University Center of Pharmaceutical Technology. The operation flow was as follows: 100 mg JCF sample was dissolved in 10 mL 50% methanol solution within a 15 mL centrifuge tube. Then 1 mL supernatant of the mixture above was centrifuged at 14,000 r/min for 5 min, with supernatant filtrating through 0.22 μm microporous membrane for UHPLC-MS/MS analysis. To analyze JCF medicated rat serum, 100 μL JCF medicated rat serum sample was mixed with 500 μL acetonitrile precipitated protein and vortexed for 2 min. And then, we took 500 μL supernatant for UHPLC-MS/MS analysis, control blank serum sample was dealt with in the same condition.

### 2.4 Detection of HBsAg and HBeAg

To detect the level of HBsAg and HBeAg in cell culture supernatant or mouse serum, we utilized the diagnostic kit for the quantitative determination of HBsAg and HBeAg with chemiluminescence ELISA quantitative detection kits (ABBOTT LABORATORIES, United States) following the manufacturer’s instructions.

### 2.5 Preparation of experimental reagent

JCF dry powder was dissolved in dimethyl sulfoxide (DMSO) to 500 mg/mL and kept at −20°C. The JCF solution was added to the cell culture with different final concentrations and DMSO as a control. We added various concentrations of JCF-medicated rat serum to the cell culture medium and used the corresponding concentration of normal rat serum as the control to observe the anti-HBV effect.

### 2.6 Histology and IHC analysis

The largest lobe of the mouse liver was fixed overnight in 4% formalin and embedded in paraffin. Liver sections were stained with H&E using a kit from Servicebio (G1003, CN). For IHC, sections were deparaffinized and rehydrated before antigen retrieval, removal of endogenous peroxidase, and blocking with normal goat serum. Then, the HBsAg (MAB-0898, MAB, CN) was added dropwise with incubation in a wet chamber overnight at 4°C. The next day, the reaction solution and HRP-labeled anti-mouse secondary antibody (GB23301, Servicebio, CN) were added dropwise and incubated at 37°C for 30 min. Diaminobenzidine (G1212, Servicebio, CN) was applied to provide a chromogen, referring to a reddish-brown color. Positive expression was defined as brown-yellow granules in the cytoplasm.

### 2.7 Cell lines

Huh7 and HEK293T cell lines were purchased from the American Type Culture Collection (Manassas, VA, United States). Professor Fengmin Lu (Peking University Health Science Center, Beijing, China) donated the HepG2, HepG2.2.15, and HepAD38 cell lines. The cells were cultured in Dulbecco’s Modified Eagle Medium (DMEM) supplemented with 10% fetal bovine serum (FBS), 100 U/mL penicillin, 100 μg/mL streptomycin, and 5% CO_2_. FBS was adjusted to 5% when JCF-medicated rat serum was utilized. HepG2.2.15 and HepAD38 cells were additionally added to the medium with 400 μg/mL G418 sulfate (InvivoGen, United States).

### 2.8 Cell viability assay

5×10^3^ cells were seeded in 96-well plates with six duplications, and after JCF or its medicated rat serum treatment, the CCK-8 assay kit (Solarbio, CN) was carried out to assess the cell viability by measuring the absorbance at the wavelength of 450 nm by the TECAN infinite M200 Multimode microplate reader (Tecan, Mechelen, Belgium).

### 2.9 Plasmids and transfection

The prcccDNA and pCMV-Cre plasmids were gifts from Professor Fengmin Lu (Peking University Health Science Center, Beijing, China). pCMV-vector was purchased from Beyotime Technology (Shanghai, China). HBx (His tag in N terminal) sequences were constructed into pCMV-vectors through KpnI and XbaI enzyme (NEB, Ipswich, MA, United States) digestion. All vectors were transfected into cells using lipofectamine 2000 (Invitrogen, Carlsbad, CA).

### 2.10 Quantitative real-time PCR

The RaPure Total RNA Mini Kit (Magen, R4011, CN) was used to extract the total RNA from the cells or tissues, and the All-in-One First-Strand Synthesis MasterMix (with dsDNase) (BioMed, BM60501S, CN) was used to reverse the process into cDNA. The Taq SYBR^®^ Green qPCR Premix (BioMed, BM60304S, CN) and Real-time PCR Detection System (Agilent Technologies, US) were used to perform a real-time quantitative polymerase chain reaction (qRT PCR). The experiments were performed in triplicate and repeated three times. The primers were provided as follows:

HBx-F: GCG​CGG​GAC​GTC​CTT​TGT​CT;

HBx-R: GTC​GGC​CGG​AAC​GGC​AGA​TG;

GAPDH-F: GGA​GCG​AGA​TCC​CTC​CAA​AAT;

GAPDH-R: GGC​TGT​TGT​CAT​ACT​TCT​CAT​GG.

### 2.11 Western blotting

The total protein was extracted from the biopsy tissue of the rat liver using the RIPA Lysis Buffer (Beyotime, P0013B, CN) according to the manufacturer’s instructions. The protein lysates were separated by 10% sodium dodecyl sulfate-polyacrylamide gel electrophoresis (SDS-PAGE) (Epizyme Biomedical Technology, PG112, CN) and then electrophoretically transferred onto the polyvinylidene fluoride membranes (Epizyme Biomedical Technology, WJ001, CN). The primary antibodies we used were rabbit anti-HBx (ABCAM, ab39716, 1:1000, United States); mouse anti-GAPDH (MBL, M171-3, 1:5000, JPN). The ImageJ software was used to analyze the integrated density of the protein bands.

### 2.12 Network pharmacological analysis of JCF

UHPLC-MS/MS analysis was used to determine the metabolites of JCF and its treated rat serum. SwissTargetPrediction and STITCH anticipated the components’ targets. From the DisGeNET, Genecards, PharmGKB, OMIM, MalaCards, and NCBI Gene databases, we gathered the disease targets of CHB. With PPI data from STRING, we constructed a pharmacological network using the Cytoscape software, and we identified key target genes using the Analyzer, CytoNCA, and MCODE plugins. We conducted Gene Ontology (GO) and KEGG analysis with David database.

### 2.13 Statistical analysis

Data for graphing was processed with GraphPad Prism 9.0 software (GraphPad Software Inc., US). Statistical analysis was performed using the SPSS 25.0 statistical software package. The data were expressed as the mean ± SD. The Student’s *t*-test was used to compare the two groups of data in the case of a normal distribution and homogeneous variance, and the one-way ANOVA was used to compare more than two groups of data. Nonparametric tests must be used if the data differs from the normal distribution. If *p* values are lower than 0.05, differences between groups are regarded as statistically significant.

## 3 Results

### 3.1 JCF contains multiple metabolites

**FIGURE 2:**
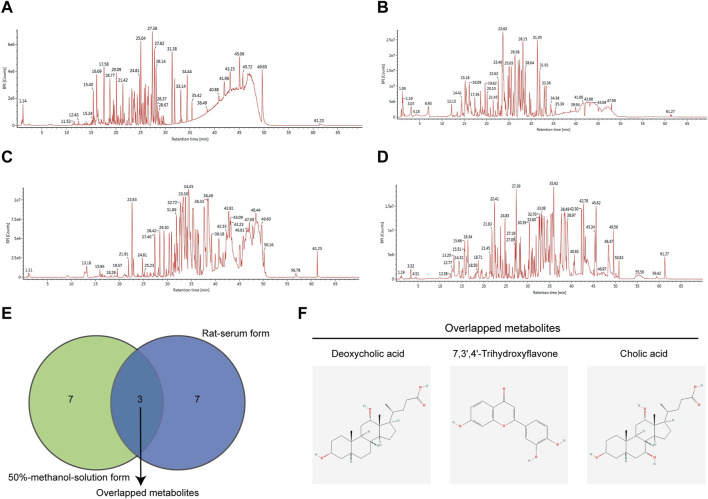
Total ion chromatography (TIC) of JCF with its 50% methanol solution form and medicated rat serum form. **(A)** TIC diagram of 50% methanol solution form of JCF in positive ion mode. **(B)** TIC diagram of 50% methanol solution form JCF in negative ion mode. **(C)** TIC diagram of JCF-medicated rat serum in positive ion mode. **(D)** TIC diagram of JCF medicated rat serum in negative ion mode. **(E)** The top 10 enriched metabolites of JCF in its 50% methanol solution form (green) and serum form (purple), respectively. **(F)** The structures of the overlapped metabolites.

First, we performed a UHPLC-MS/MS study on the 50% methanol solution and the medicated rat serum forms of JCF. Figure 2 depicts the total ion chromatography (TIC) diagram, which displays the positive and negative ion modes for the 50% methanol solution form (Figures 2A, B) and the medicated rat serum form (Figures 2C, D), respectively. The findings revealed that 23 metabolites and 92 metabolites were identified in the JCF-mediated rat serum and 50% methanol solution, respectively. The details are provided in Supplementary Tables S2, S3, respectively. We defined the metabolites with a content percentage ranking of 10 as the main metabolites. Among them, the top 10 of the 50% methanol solution of JCF were Cholic Acid (14.781%), Deoxycholic Acid (7.332%), 3′,4′,7-Trihydroxyflavone (5.759%), Gallic Acid (4.201%), Pectolinarigenin (4.126%), Oleanolic Acid (4.126%), Betulinic Acid (3.812%), Soyasaponin I (2.35%), Ginsenoside Rb1 (2.346%), and (20R)-Ginsenoside Rg3 (2.318%), and the information was shown in Table 1. The top 10 of JCF medicated rat serum were Cholic Acid (47.775%), Linoleic Acid (21.238%), Deoxycholic Acid (16.223%), Elaidic Acid-1 (7.327%), Hexadecanoic Acid (4.155%), Riboflavin (0.769%), Geniposide (0.556%), Ambrettolide (0.489%), 3′,4′,7-Trihydroxyflavone (0.374%), and Panaxydol (0.177%), and the information was shown in Table 2. We discovered that JCF and its medicated rat serum had a definite association. Cholic Acid, Deoxycholic Acid, and 3′,4′,7-Trihydroxyflavone were the key metabolites in both (Figure 2E). We hypothesized that 3′,4′,7-Trihydroxyflavone, Cholic Acid, and Deoxycholic Acid could be the main active metabolites in JCF, and their structures were shown in Figure 2F.

**TABLE 1:** The information on the main metabolites in the 50%-methanol-solution form of JCF.

Metabolite name	CAS number	Formula	Response	Neutral Mass (Da)	Mass error (ppm)	Observed RT (min)	Adducts	Relative amounts (%)
Cholic Acid	81–25–4	C24H40O5	2916780	408.2876	1.5	27.4	-H	14.781
Deoxycholic Acid	83–44–3	C24H40O4	2386508	392.2927	1.8	28.15	-H	7.332
3′,4′,7-Trihydroxyflavone	2150–11–0	C15H10O5	1183779	270.0528	−4.7	18.78	+H	5.759
Gallic Acid	149–91–7	C7H6O5	929866	170.0215	1	6.93	-H	4.201
Pectolinarigenin	520–12–7	C17H14O6	678236	314.079	−3.5	26.45	+H	4.126
Oleanolic Acid	508–02–1	C30H48O3	666206	456.3604	1.6	48.02	-H	4.126
Betulinic Acid	472–15–1	C30H48O3	666206	456.3604	1.6	48.02	-H	3.812
Soyasaponin I	51330-27-9	C48H78O18	615439	942.5188	2.5	26.61	+H	2.35
Ginsenoside Rb1	41753–43–9	C54H92O23	379464	1108.603	4.4	23.55	-H	2.346
(20R)-Ginsenoside Rg3	38243–03–7	C42H72O13	378793	784.4973	0.5	29.64	-H	2.318

**TABLE 2:** The information on the main metabolites in the serum form of JCF.

Metabolite name	CAS number	Formula	Response	Neutral Mass (Da)	Mass error (ppm)	Observed RT (min)	Adducts	Relative amounts (%)
Cholic Acid	81–25–4	C24H40O5	2605858	408.2876	1.1	27.41	-H	47.775
Linoleic Acid	60–33–3	C18H32O2	1158415	280.2402	1.4	43.16	-H	21.238
Deoxycholic Acid	83–44–3	C24H40O4	884,865	392.2927	0.7	28.15	-H	16.223
Elaidic Acid-1	112–79–8	C18H34O2	399,624	282.2559	0.8	45.72	-H	7.327
Hexadecanoic Acid	57–10–3	C16H32O2	226,615	256.2402	1.7	45.07	-H	4.155
Riboflavin	83–88–5	C17H20N4O6	41,943	376.1383	−0.7	15.4	+H, +Na	0.769
Geniposide	24512–63–8	C17H24O10	30,307	388.137	−2.5	15.16	+Na	0.556
Ambrettolide	123–69–3	C16H28O2	26,669	252.2089	2.4	28.15	+Na	0.489
3′,4′,7-Trihydroxyflavone	2150–11–0	C15H10O5	20,418	270.0528	−6.9	18.8	+H	0.374
Panaxydol	72800–72–7	C17H24O2	9648	260.1776	−7.1	31.4	+H	0.177

### 3.2 JCF accelerates the clearance of HBsAg in HBV replicating mouse model

**FIGURE 3:**
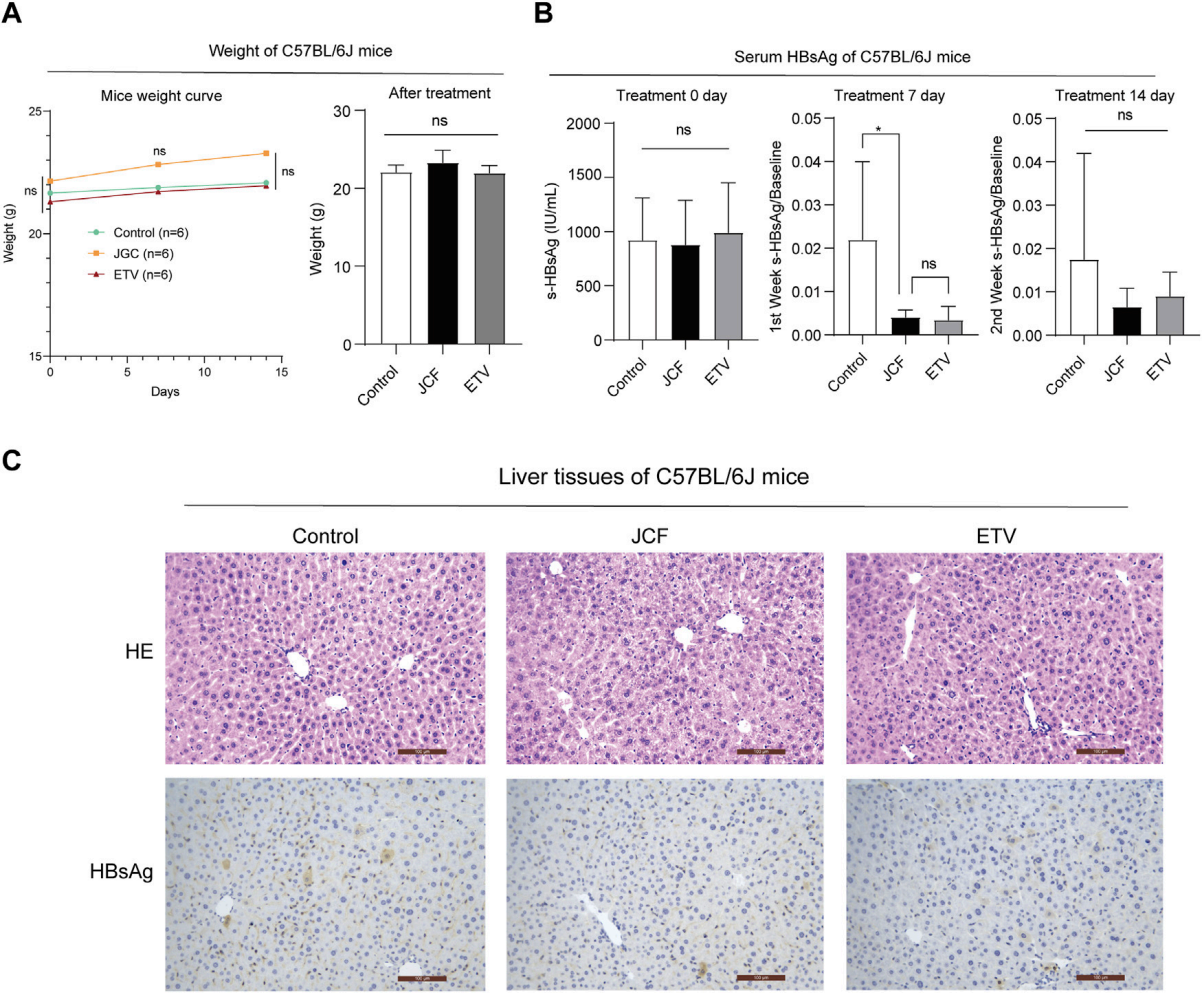
JCF accelerates the elimination of serum HBsAg and associated intrahepatic deposits in HBV replication C57BL/6J mice. **(A)** C57BL/6J mice weights at days 0, 7, and 14 after HDI, *n* = 6 in each group. **(B)** C57BL/6J mice’s serum HBsAg levels at days 0, 7, and 14 after treatment, *n* = 6 in each group. **(C)** The representative images of H&E staining and HBsAg IHC of mice’s liver. The images are presented at high power (× 200, Scale bars = 100 μm). *p < 0.05 versus the Control group. ns, not significant.

We next conducted an *in vivo* experiment using an HBV replicating mouse model constructed via hydrodynamic tail vein injection of HBV plasmids to confirm the role of JCF on anti-HBV. The ETV was taken as a positive control. After plasmid injection, mice received clinically equivalent dosages of JCF and ETV via gavage for 2 weeks. There was no significant difference in mice weight in each group during the modeling process and at the end of the experiment (Figure 3A). On the first day after the model was established, the mice’s serum HBsAg levels were around 1000 U/mL, and there was no significant difference between groups (Figure 3B left). The serum HBsAg levels of the mice in the JCF and ETV treatment groups were significantly lower after 7 days of treatment than those in the Control group (Figure 3B middle). The serum HBsAg level of mice dropped to around 0.02 IU/mL after 14 days of treatment with medication, and there was no statistically significant difference between groups (Figure 3B right). Additionally, we utilized H&E staining and HBsAg IHC to identify the liver pathology in each group of mice. The results revealed that all of the mice had normal liver pathology at the end of the experiment and that JCF and ETV treatment significantly lowered HBsAg expression compared to the Control (Figure 3C). These experimental findings showed that JCF therapy might accelerate HBsAg elimination in HBV replication mice.

### 3.3 JCF suppresses the replication of HBV *in vitro*

**FIGURE 4:**
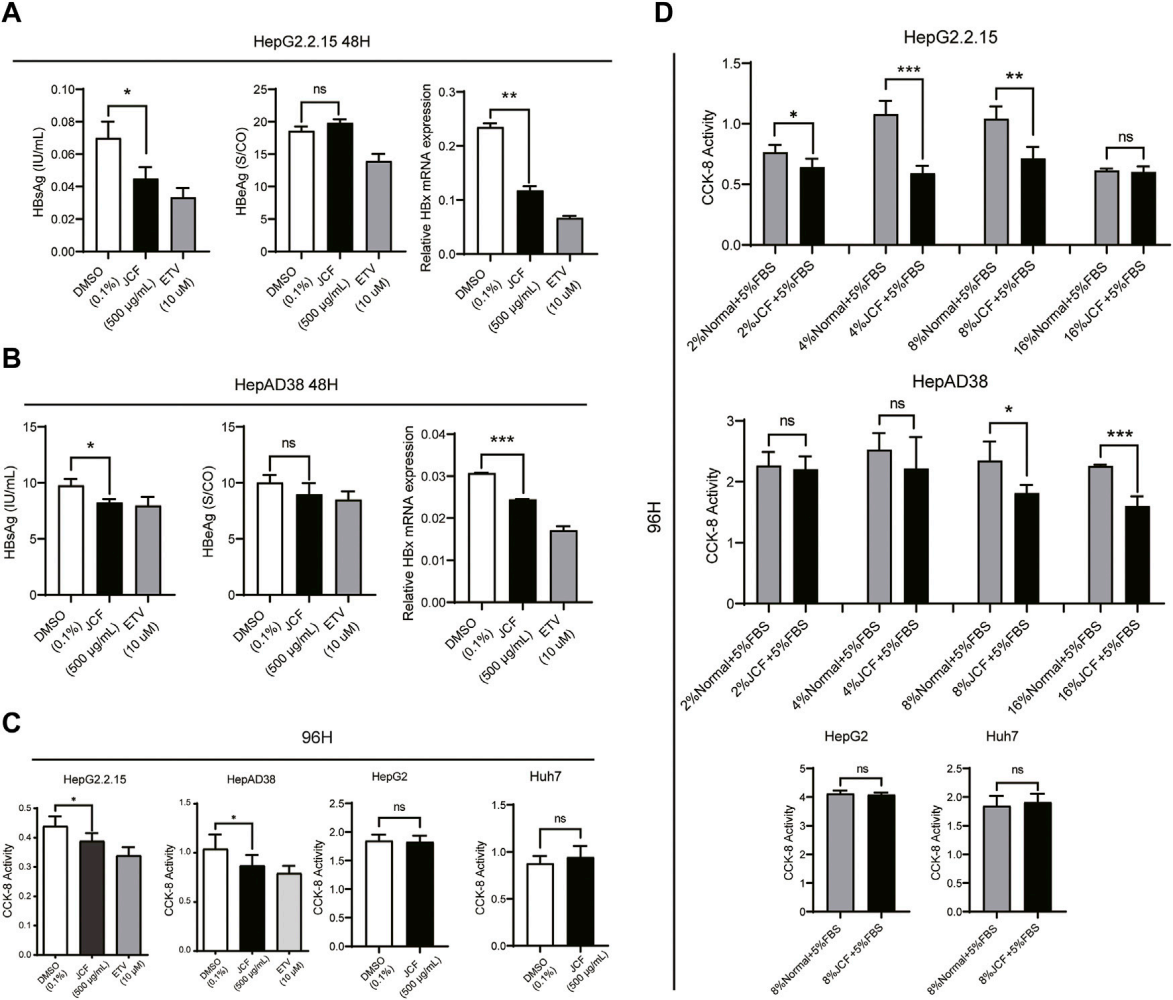
JCF inhibits the replication of HBV and the proliferation of HBV-related hepatoma cells *in vitro*. **(A, B)** HepG2.2.15 **(A)** and HepAD38 **(B)** cells were treated with DMSO (0.1%), JCF (50 0 μg/mL), and ETV (10 μM) for 48 h. The levels of HBsAg and HBeAg in cell supernatants were examined by ELISA, and the mRNA expression levels of HBx were detected by qRT-PCR. **(C)** CCK-8 assays were used to determine the effects of DMSO (0.1%) and JCF (500 μg/mL) on the proliferation of HepG2.2.15, HepAD38, HepG2, and Huh7 cell lines after 96 h of treatment. **(D)** CCK-8 assays were used to determine the effects of gradient concentration JCF medicated rat serum on the proliferation of HepG2.2.15, HepAD38 cells, HepG2, and Huh7 cells after 96 h of treatment. Data are presented as the mean ± SD, and the *p*-value was calculated by one-way ANOVA between 3 groups and Student’s t-test between 2 groups. *p < 0.05, **p < 0.01, ***p < 0.001. ns, not significant.

To investigate if JCF had a direct anti-HBV impact, we subsequently treated HepG2.2.15 and HepAD38 cells with 500 g/mL JCF for 48 h. As negative and positive controls, we used 0.1% DMSO and 10 mM ETV. JCF and ETV considerably decreased the quantities of HBsAg in supernatants and the mRNA levels of HBx in both HepG2.2.15 (Figure 4A) and HepAD38 (Figure 4B) cells as compared to the negative control. We next administered the medications to HepG2.2.15, HepAD38, HepG2, and Huh7 cells for 96 h and tested the vitality of the cells, considering that HBV replication is correlated with the proliferation of hepatoma cells (Chuang et al., 2022). HepG2.2.15 and HepAD38 cell proliferation was decreased by JCF and ETV treatment in comparison to the negative control, whereas HepG2 and Huh7 cells were unaffected (Figure 4C). Consistent with the above results, with JCF-medicated rat serum, we also found that JCF-medicated rat serum inhibited the proliferation of HepG2.2.15 and HepAD38 cells and did not affect the proliferation of HepG2 and Huh7 cells (Figure 4D). These results showed that JCF could exert a direct suppressive effect on HBV replication in cells, further inhibiting the proliferation of HBV-related hepatoma cells.

### 3.4 JCF suppresses the replication of HBV relying on HBx expression

**FIGURE 5:**
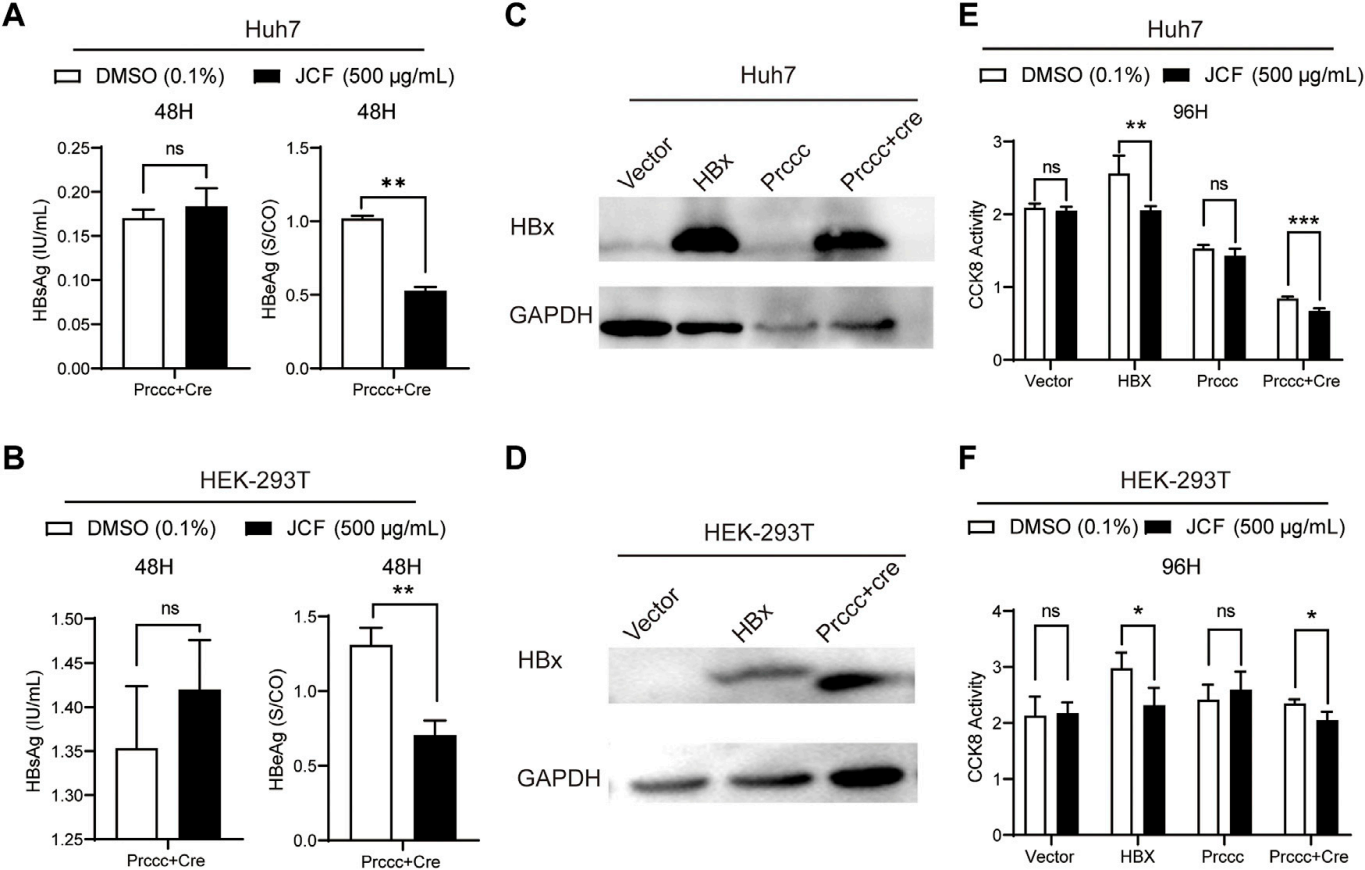
JCF inhibits HBx-expressed cells’ proliferation. **(A, B)** Huh7 **(A)** and HEK-293T **(B)** cells were transfected with HBV-expressing plasmids for 48 h and then treated with DMSO and JCF for another 48 h. The levels of HBsAg and HBeAg in the supernatants were examined by ELISA. **(C, D)** Huh7 **(C)** and HEK-293T **(D)** cells were transfected with HBx and HBV-expressing plasmids for 48 h, respectively. The HBx expression was detected by Western blotting. **(E, F)** Huh7 **(E)** and HEK-293T **(F)** cells were transfected with HBx and HBV-expressing plasmids for 48 h and then treated with DMSO and JCF for another 96 h. The cells’ viability was detected by CCK-8. Data are presented as the mean ± SD, and the *p*-value was calculated by the Student’s T-test between the 2 groups. *p < 0.05, **p < 0.01, ***p < 0.001. ns, not significant.

We conducted experiments using HBV plasmids transfected into no-HBV cell lines of Huh7 and HEK-293T cells to further support the inhibitory impact of JCF on HBV. The outcomes demonstrated that JCF considerably suppresses HBeAg expression in these transfecting cells (Figures 5A, B). We then conducted experiments with HBx and HBV plasmids transfected into no-HBV cell lines of Huh7 and HEK-293T cells, respectively, taking into account that HBx is a crucial factor to govern HBV replication and the proliferation of hepatoma cells (Lucifora et al., 2011). And we found that in Huh7 and HEK-293T cells with HBx overexpressed (Figures 5C, D), JCF could significantly inhibit cells’ proliferation (Figures 5E, F). In conclusion, these results indicated that JCF might inhibit the replication of HBV in an HBx-reliant manner.

### 3.5 The network pharmacological results of JCF on CHB treatment

**FIGURE 6:**
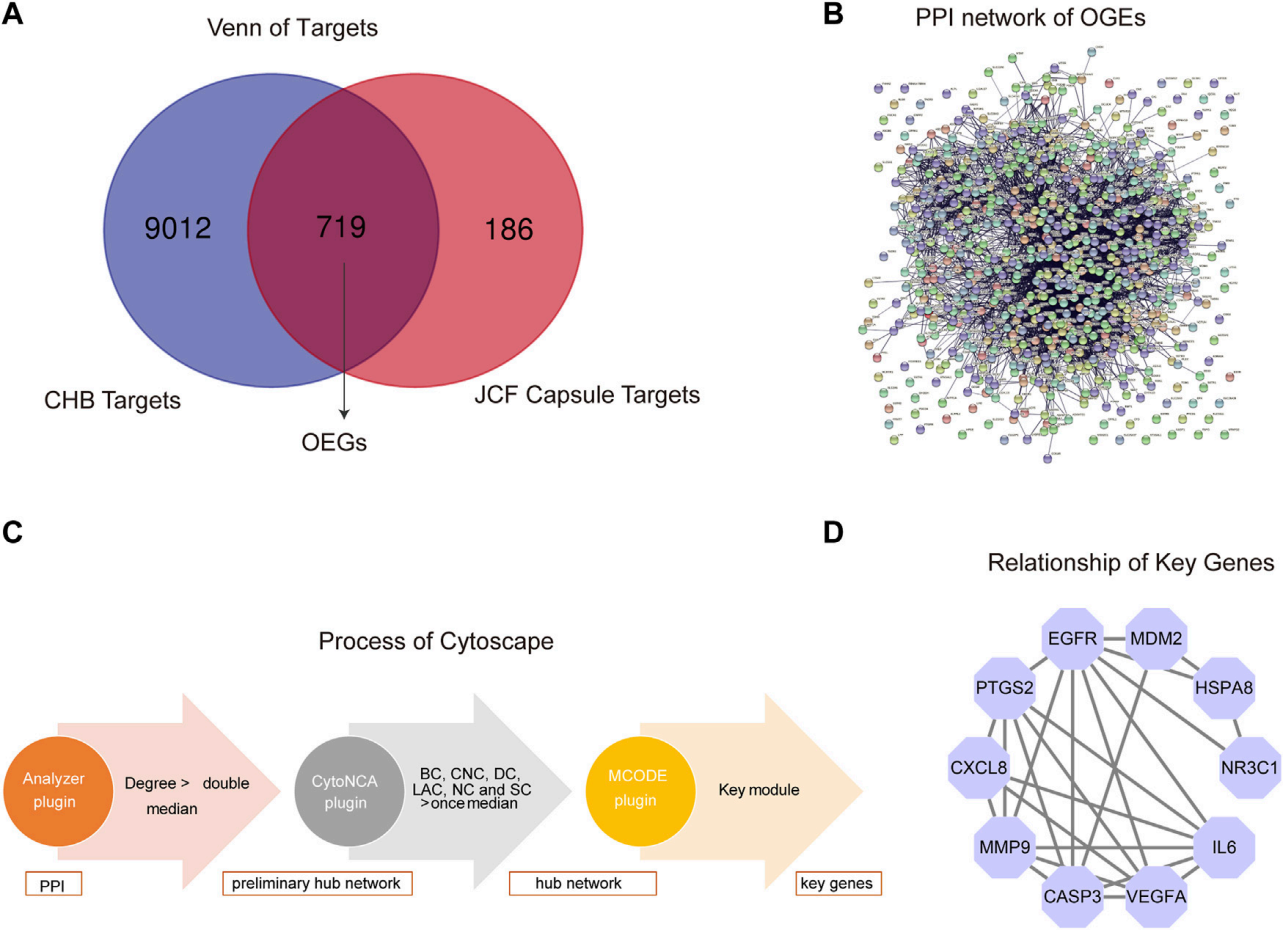
Network pharmacological analysis of JCF on CHB treatment. **(A)** The OGEs of JCF on CHB treatment. The blue circle represents CHB targets, and the red circle represents JCF putative targets. **(B)** The PPI network of OGEs in the STRING database. **(C)** Process of network pharmacological analysis using Cytoscape software based on the topological method. **(D)** The relationship of key genes, the purple circles represent key target genes, and the black line indicates their relationship.

We also carried out network pharmacological analysis to better understand the mechanism of JCF in treating CHB. Firstly, according to UHPLC-MS/MS analysis of the JCF, SwissTarget Prediction identified 663 target genes and STITCH identified 292 target genes. After removing repeated genes, a final total of 905 target genes was identified. Supplementary Table S4 displays detailed information on target genes. Then, a total of 8879 targets were found in the GeneCards database, 415 targets in the DisGeNET database, 1038 targets in the OMIM database, 253 targets in the NCBI Gene database, 58 targets in the MalaCards database, and 39 targets in the PharmGKB database, respectively, to acquire the disease target genes of CHB. And a total of 9731 overlapped disease target genes were obtained. Detailed information on disease target genes was shown in Supplementary Table S5. Besides, using the Venn diagrams tool, we uploaded the potential targets of the JCF and the disease targets of the CHB and extracted 719 overlapped genes (OGEs) (Figure 6A). We constructed a protein-protein interaction (PPI) network using the STRING database and adjusted the necessary interaction score to 0.7 to further investigate the underlying mechanism of these OGEs (Figure 6B). The PPI network was then analyzed using Cytoscape’s Analyzer plugin to create a preliminary hub network based on Degree >20. The preliminary hub network was then analyzed using the CytoNCA plugin to produce a hub network that conformed to the following standards: Subgraph centrality (SC) > 1.584E+13, Betweenness Connectivity (BC) > 0.005, Closeness Connectivity (CC) > 0.382, Degree Connectivity (DC) > 31, Local Average Connectivity (LAC) > 11.125, Neighbor Connectivity (NC) > 38.565. To determine the main module and genes, we also calculated the hub network using the MCODE plugin (Figure 6C). The important genes were CASP3, CXCL8, EGFR, HSPA8, IL6, MDM2, MMP9, NR3C1, PTGS2, and VEGFA, and Figure 6D showed the relationship between them.

### 3.6 The potential pathways regulated by JCF

**FIGURE 7:**
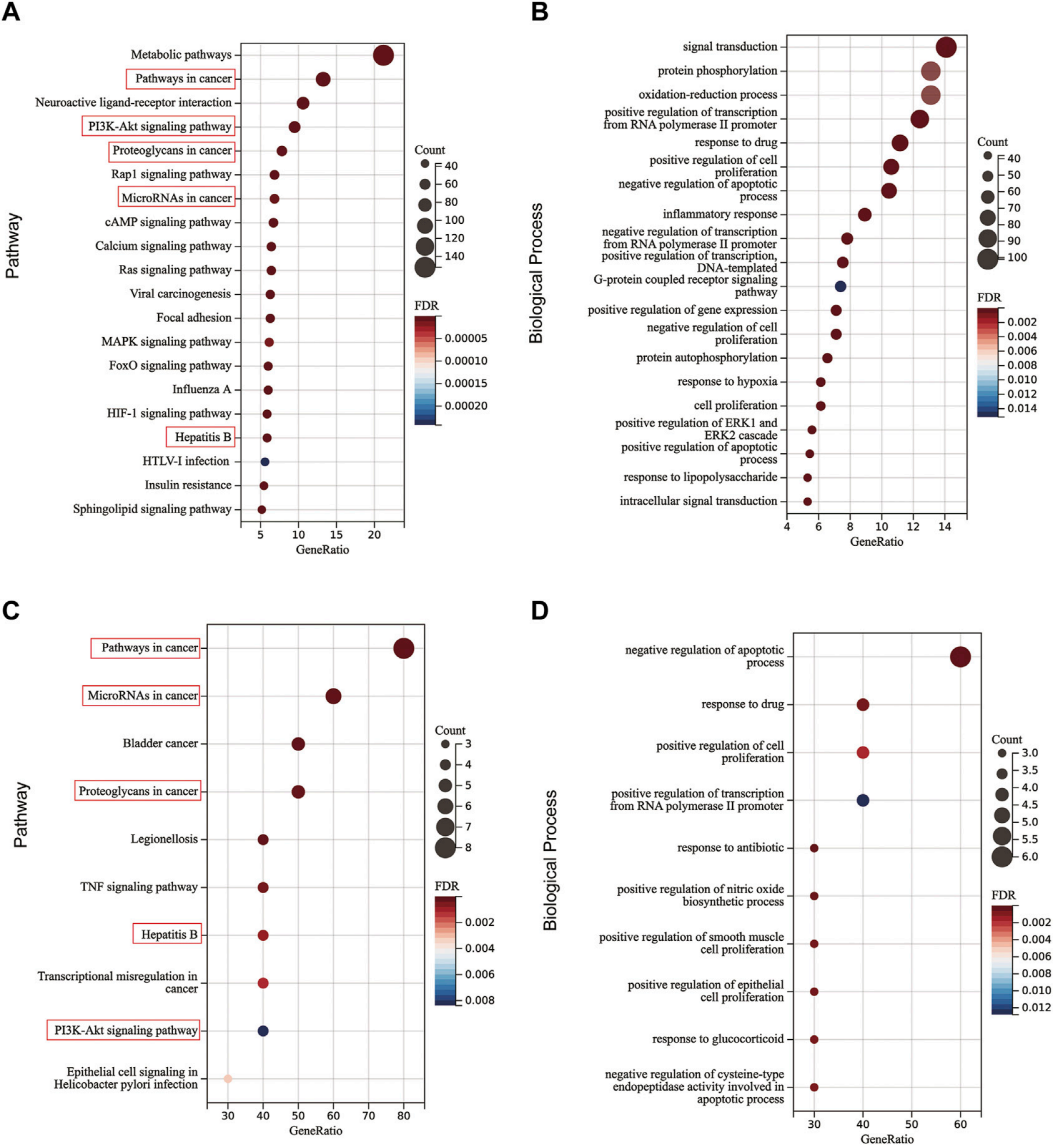
The KEGG and BP functional enrichment of OGEs and key genes. **(A)** Enriched KEGG pathways of OGEs. **(B)** Enriched GO-BP of OGEs. **(C)** Enriched KEGG pathways of key genes. **(D)** Enriched GO-BP of key genes. The red box represents the repetitive key pathways.

To further elucidate the potential pathways regulated by JCF, a functional enrichment analysis of JCF’s putative genes and key genes was performed. The KEGG enrichment analysis suggested that JCF’s putative target genes were primarily associated with metabolic pathways, pathways in cancer, neuroactive ligand-receptor interaction, etc. (Figure 7A). And the GO enrichment analysis results showed that the enriched biological process (BP) of JCF’s putative target genes was primarily correlated to signal transduction, protein phosphorylation, the oxidation-reduction process, etc. (Figure 7B). For the key targets we obtained, the KEGG enrichment results suggested that they were mostly involved in pathways of cancer, microRNAs in cancer, bladder cancer, etc. (Figure 7C). And the GO enrichment analysis results showed that the key genes were significantly related to multiple GO-BP, including negative regulation of the apoptotic process, response to drugs, positive regulation of cell proliferation, etc. (Figure 7D). And the detailed information is shown in Supplementary Table S6. According to the above findings, pathways in cancer, hepatitis B, microRNAs in cancer, PI3K-Akt signaling, and proteoglycans in cancer were all enriched in both the putative and key targets. Generally speaking, JCF might regulate pathways related to CHB. These biological processes and signaling pathways may be connected to JCF’s protective effects against CHB.

## 4 Discussion

According to clinical studies, JCF might enhance the anti-HBV efficacy of ETV by lowering levels of ALT, AST, total bilirubin, laminin, collagen type IV (IV-c), and type-III procollagen (Lei, 2018b). Additionally, JCF combination therapy could improve aberrant liver function and had higher efficacy than standard treatment (Lei, 2018a). In basic terms, our study used UHPLC-MS/MS to identify the key metabolites of JCF, and network pharmacology combined with *in vivo* and *in vitro* experiments was used to investigate the underlying mechanism of JCF in treating CHB.

Based on UHPLC-MS/MS findings, 3′,4′,7-Trihydroxyflavone, Cholic Acid, and Deoxycholic Acid may be the main metabolites of JCF in the treatment of CHB. Cholic acid is critical for lipid metabolism, vitamin absorption, liver function, and enterohepatic circulation, according to earlier research (Guzior and Quinn, 2021). Deoxycholic acid is a natural secondary hydrophilic cholic acid, which can prevent the formation of gallstones (Song et al., 2020). Cholic acid-nucleoside analog conjugates can enhance the uptake of antiviral drugs by hepatocytes and increase the bioavailability of oral drugs (Li et al., 2019). 3′,4′,7-Trihydroxyflavone has protective effects against acute liver injury in mice via alleviating inflammatory response, oxidative, and nitrosative stress burden, whose mechanism is about activating the Nrf2 and suppressing the TLR4/NF-κB signaling pathways (Islam et al., 2019). In addition, the metabolites of JCF’s Geniposide and Ginsenoside Rb1 demonstrated significant anti-inflammatory and liver-protective characteristics. TNF and IL-1 are examples of pro-inflammatory cytokines that can be decreased by ginsenoside Rb1 (Lai et al., 2021). According to a previous study (Fan et al., 2020), Geniposide might regulate pyrolysis, necrosis, apoptosis, and autophagic cell death. Further research is required to fully understand how these metabolites work and how they’re employed to treat CHB.

Our KEGG enrichment analysis revealed the mechanism of JCF in CHB therapy could have a connection between hepatitis B and cancer-related pathways. The Hepatitis B pathway’s main targeted genes are IL-6, CXCL8, CASP3, and MMP9. IL-6 is the main regulator in balancing Treg cells and Th17 cells, which are closely related to CHB, HBV-related chronic and acute liver failure (ACLF), and HCC(Taniguchi and Karin, 2014). And the REVEAL-HBV study has reported that the increased risk of HCC was related to the upregulation of IL-6 levels (Koshiol et al., 2021). The primary biological function of CXCL8 is neutrophil recruitment and activation (Brugman et al., 2014). Based on study findings, HBx can trigger liver inflammation by increasing the production of the pro-inflammatory cytokines IL-6, CXCL8, and CXCL2 in hepatocytes (Luo et al., 2015). Hepatitis B has a strong connection with CASP3, an effector protein of the caspase family that can cause the nuclear DNA to increase during the apoptotic process (Ma et al., 2021). It has been noted that MMP-9 inhibits the IFN/JAK/STAT pathway, which promotes HBV replication (Chen et al., 2017). Furthermore, by binding to IFNAR1, MMP-9 may stimulate IFN alpha receptor 1 (IFNAR1) phosphorylation, ubiquitination, subcellular distribution, and degradation. Additionally, MMP-9 might inhibit IFANR1’s capacity to bind to IFN-α, therefore suppressing the innate immune response in hepatocytes (Chen et al., 2017). We hypothesized that JCF would slow the progression of CHB by lowering liver inflammation, recovering liver immune function, and regulating the expression of key genes in the Hepatitis B pathway. However, further study in infectious animal models is required.

In addition, our results showed that JCF could also inhibit the proliferation of HBV-related hepatoma cells via an HBx-dependent manner, and the key genes were enriched for cancer-related pathways, including the PI3K-Akt pathway, proteoglycans in cancer, microRNAs in cancer, and pathways in cancer. We discovered that MDM2, PTGS2, EGFR, and VEGFA are the key genes regulated by JCF and closely associated with the development of cancer. Studies have shown that HBx might increase MDM2 expression (Wang et al., 2017). Also, HBV infection may cause the MDM2/p53 axis to become dysfunctional, which presents as p53 inactivation and MDM2 overactivation and results in HCC (Cao et al., 2020). In addition, HBx might increase the activity of Cyclooxygenase-2 (COX-2, PTGS2), which leads to tumor cell invasion, and activate both MT1-MMP expression and cell invasion (Lara-Pezzi et al., 2002). Furthermore, HBx-induced mitochondrial damage raises ROS levels and COX-2 gene expression, which increases liver inflammation (Yoo et al., 2019). The intrinsic sodium taurocholate co-transporting polypeptide (NTCP)-EGFR complex is what drives HBV into human hepatocytes and is a necessary entry cofactor for HBV infection (Iwamoto et al., 2019). It was reported that small HBV surface antigens (SHBs) enhanced the angiogenic capacity of HCC cells by inducing endoplasmic reticulum (ER) stress, which consequently activated unfolded protein response (UPR) signaling to increase VEGFA expression and secretion (Wu et al., 2022). According to the experimental results, our study suggested that JCF may prevent the development of HBV-related HCC by inhibiting the replication of HBV and reducing the expression of HBx. The results require additional confirmation since we hypothesized that the suppression of HBV replication correlates with the control of immunity.

In summary, JCF’s main metabolites and related signaling pathways for treating CHB have been identified using network pharmacology and UHPLC-MS/MS analysis. Combining the HBV replication mouse model and cell experiments, it was hypothesized that JCF might regulate pathways associated with inflammation and enhance the immune response to exert anti-HBV activity by directly reducing HBsAg and HBx expression. In addition, we investigated several potential pharmacological ingredients that could help in JCF’s complementary and alternative therapies. This research also comes with a few restrictions. First of all, more experiments are required to support the anti-HBV activities of the 3 key active metabolites of JCF. Next, further study is required to determine how the JCF interacted with the key targets we obtained to block HBV. Finally, the HBV infection animal and cell models are very necessary in further study.

## 5 Conclusion

JCF exerted functions of anti-HBV *in vivo* and *in vitro* experiments and inhibited the proliferation of HBV-replicating hepatoma cell lines. The JCF key metabolites were 3′,4′,7-Trihydroxyflavone, Cholic Acid, and Deoxycholic Acid, and the mechanism might be related to pathways in cancer, hepatitis B, microRNAs in cancer, PI3K-Akt signaling, and proteoglycans in cancer pathways. And our research has provided a wider basis and support for JFC’s clinical applications.

## Figures and Tables

**Table:** 

**ACLF**	acute liver failure
**ALT**	alanine-transaminase
**AST**	aspartate aminotransferase
**BC**	Betweenness Connectivity
**BP**	biological process
**CC**	Closeness Connectivity
**cccDNA**	covalently closed circular DNA
**CHB**	chronic hepatitis B
**COX-2**	Cyclooxygenase-2
**DC**	Degree Connectivity
**DMEM**	Dulbecco’s modified eagle medium
**DMSO**	dimethyl sulfoxide
**ER**	endoplasmic reticulum
**ETV**	entecavir
**FBS**	fetal bovine serum
**GO**	Gene Ontology
**HBeAg**	HBV e antigen
**HBsAg**	HBV surface antigen
**HBV**	hepatitis B virus
**HBx**	HBV x
**HCC**	hepatocellular carcinoma
**HDI**	hydrodynamic injection
**H&E**	hematoxylin and eosin
**IFNAR1**	interferon alpha receptor 1
**IFNs**	interferons
**IHC**	immunohistochemical
**IV-c**	collagen type IV
**JCF**	JiGuCao capsule formula
**LAC**	Local Average Connectivity
**LN**	laminin
**MS**	mass spectrometry
**NAs**	nucleoside analogues
**NC**	Neighbor Connectivity
**NTCP**	sodium taurocholate cotransporting polypeptide
**OGEs**	overlapped genes
**PBS**	phosphate buffered saline
**pg RNA**	pregenomic RNA
**PPI**	protein-protein interaction
**qRT-PCR**	quantitative Real-time PCR
**SC**	Subgraph Centrality
**SHBs**	small HBV surface antigen
**SPF**	specific pathogen-free
**TBil**	total bilirubin
**TCM**	Traditional Chinese medicine
**TIC**	total ion chromatography
**UHPLC-MS**	ultra-high performance liquid chromatography-tandem mass spectrometry
**UPR**	unfolded protein response
**WHO**	World Health Organization

## Data Availability

The datasets presented in this study can be found in online repositories. The names of the repository/repositories and accession number(s) can be found in the article/Supplementary Material.
